# The temperature dependence of maltose transport in ale and lager strains of brewer's yeast

**DOI:** 10.1111/j.1567-1364.2010.00627.x

**Published:** 2010-04-09

**Authors:** Virve Vidgren, Jyri-Pekka Multanen, Laura Ruohonen, John Londesborough

**Affiliations:** VTT Technical Research Centre of FinlandEspoo, Finland

**Keywords:** brewer's yeast strains, evolution, α-glucoside transporters, temperature dependence of fermentation, temperature dependence of transport

## Abstract

Lager beers are traditionally made at lower temperatures (6–14 °C) than ales (15–25 °C). At low temperatures, lager strains (*Saccharomyces pastorianus*) ferment faster than ale strains (*Saccharomyces cerevisiae*). Two lager and two ale strains had similar maltose transport activities at 20 °C, but at 0 °C the lager strains had fivefold greater activity. *AGT1, MTT1* and *MALx1* are major maltose transporter genes. In nine tested lager strains, the *AGT1* genes contained premature stop codons. None of five tested ale strains had this defect. All tested lager strains, but no ale strain, contained *MTT1* genes. When functional *AGT1* from an ale strain was expressed in a lager strain, the resultant maltose transport activity had the high temperature dependence characteristic of ale yeasts. Lager yeast *MTT1* and *MALx1* genes were expressed in a maltose-negative laboratory strain of *S. cerevisiae*. The resultant Mtt1 transport activity had low temperature dependence and the Malx1 activity had high temperature dependence. Faster fermentation at low temperature by lager strains than ale strains may result from their different maltose transporters. The loss of Agt1 transporters during the evolution of lager strains may have provided plasma membrane space for the Mtt1 transporters that perform better at a low temperature.

## Introduction

Among different beer types, ales are made by fermentation of brewer's wort at 15–25 °C, whereas lagers are traditionally made by fermentation at lower temperatures, 6–14 °C (Bamforth, 1998). Brewer's yeasts can be divided into ale strains (which cannot use melibiose) and lager strains (which can). Ale strains usually grow and ferment poorly at temperatures below about 12 °C, whereas lager strains perform well down to at least 7 °C (Walsh & Martin, 1977). Ale strains have been used for thousands of years, but it has been suggested that lager strains probably originated in low-temperature wort fermentations in Bavaria a few hundred years ago (see, e.g. Hornsey, 2003).

Most ale strains are thought to be varieties of *Saccharomyces cerevisiae* (Hammond, 1993; Tornai-Lehoczki & Dlauchy, 2000; Kobi *et al.*, 2004;). However, it has been shown recently that they include strains (e.g. isolates from Trappist beers) that are hybrids between *S. cerevisiae* and *Saccharomyces kudriavzevii* (Gonzalez *et al.*, 2008) and some other ale strains previously classified as *S. cerevisiae* may be hybrids (Querol & Bond, 2009). Lager strains have been variously described as *Saccharomyces carlsbergensis, Saccharomyces uvarum* and *Saccharomyces pastorianus* (Hammond, 1993). They have alloploid genomes and are hybrids of two species: *S. cerevisiae* and *Saccharomyces bayanus* (Naumova *et al.*, 2005; Caesar *et al.*, 2007; Dunn & Sherlock, 2008;). The hybrids are thought to have been selected during low-temperature wort fermentations. The cryophilic nature of lager yeasts probably derives from the *S. bayanus* partner (Sato *et al.*, 2002). The complete sequencing of lager strain WS34/70 confirmed its hybrid nature: 36 chromosomes were found, 16 of *S. cerevisiae* type, 12 of *S. bayanus* type and 8 chimeric (Nakao *et al.*, 2009). The presence of the chimeric chromosomes (part *S. cerevisiae*, part *S. bayanus*) suggested posthybridizational reorganization. Dunn & Sherlock (2008) also reported posthybridizational reorganization of lager strain chromosomes, and divided lager strains into two groups, originating from two separate hybridization events between *S. cerevisiae* and *S. bayanus*. They propose that distinct, but similar, *S. cerevisiae* strains were involved in the two events, and that hybridization was followed by the loss of a large portion of the *S. cerevisiae* genome from Group 1, whereas in Group 2, the loss of *S. cerevisiae* genes was much smaller.

The α-glucosides maltose and maltotriose together account for about 80% of the total fermentable sugars in brewer's wort. Efficient assimilation of these sugars is essential for fast and complete wort fermentations. All yeast α-glucoside transport systems characterized so far are H^+^-symporters that are driven by the electrochemical proton gradient across the plasma membrane. The active transport of maltose and maltotriose across the plasma membrane is a major rate-limiting step in the fermentation of wort (Kodama *et al.*, 1995; Rautio & Londesborough, 2003; Stambuk *et al.*, 2006;).

Several kinds of genes for α-glucoside transporters are found in *Saccharomyces* yeasts. *MALx1* genes (*x*=1–4 and 6) occur in five unlinked *MAL* (maltose) loci (*MAL1*–*MAL4* and *MAL6*). Most studies (e.g. Han *et al.*, 1995; Salema-Oom *et al.*, 2005; Alves *et al.*, 2008;) indicate that the Malx1 transporters are narrowly specific for maltose (*K*_m_∼3 mM) and turanose, but activity towards maltotriose has been claimed (Day *et al.*, 2002a). The identical *MPH2* and *MPH3* (**m**altose **p**ermease **h**omologue) occur on different chromosomes and encode transporters able to carry maltose (*K*_m_∼4 mM), maltotriose (*K*_m_∼7 mM), α-methylglucoside and turanose (Day *et al.*, 2002b). *AGT1* (**α**-**g**lucoside **t**ransporter) encodes the α-glucoside transporter with the widest substrate specificity reported so far (Han *et al.*, 1995). The Agt1 transporter can carry trehalose and sucrose (*K*_m_∼8 mM) as well as maltose, maltotriose and α-methylglucoside (*K*_m_ 20–35 mM) (Stambuk & de Araujo, 2001). A gene called *MTT1* (**mt**y-like **t**ransporter; Dietvorst *et al.*, 2005) or *MTY1* (**m**altotriose **t**ransport in **y**east; Salema-Oom *et al.*, 2005) encodes a transporter that has a higher affinity for maltotriose (*K*_m_∼20 mM) than for maltose (*K*_m_∼70 mM) and can also carry trehalose and possibly turanose (Salema-Oom *et al.*, 2005). Nakao *et al.* (2009) have recently sequenced the genome of the lager strain, Weihenstephan 34/70. They found a gene, LBYG13187, that they believe to be the *S. bayanus* counterpart of the *S. cerevisiae AGT1* because its closest homology was 79% identity to the *AGT1* sequence in the *Saccharomyces* genome database (SGDB, where *AGT1* is referred to as *MAL11*). Here, we call this gene *Sb-AGT1*, although nothing is known yet about the substrate specificity or other properties of the transporter it encodes.

Several authors have compared maltose transport by individual ale and lager yeast strains. For example, Crumplen *et al.* (1996) found that glucose more strongly inhibited maltose transport by an ale strain than that by a lager strain. Rautio & Londesborough (2003) found that trehalose and sucrose (a substrate of only Agt1 among known maltose transporters) strongly inhibited maltose transport by an ale strain, but only weakly inhibited maltose transport by a lager strain. Vidgren *et al.* (2005) reported that α-methyl glucoside (a substrate of only Agt1 and Mphx transporters) inhibited maltose transport into two ale strains by 41–74%, but inhibited maltose transport into three lager strains by only 10–23%. Taken together, these results suggested that the dominant maltose transporters of the ale strains studied had a broader specificity than those of lager strains, and were probably Agt1 proteins.

However, hybridization studies showed that all the ale and lager strains tested contained *AGT1* and several *MALx1* genes (Jespersen *et al.*, 1999; Vidgren *et al.*, 2005;). This apparent discrepancy was partially resolved by the finding (Vidgren *et al.*, 2005) that the *AGT1* genes of two lager strains contained premature stop codons. The same defect has been found in other lager strains, but not in ale strains (Vidgren *et al.*, 2007; Nakao *et al.*, 2009;). *MTT1* genes were found in all four lager strains examined by Dietvorst *et al.* (2005). *MPHx* sequences were found (usually on chromosome IV, corresponding to *MPH2*) in some, but not all lager strains and less frequently in ale strains (Jespersen *et al.*, 1999; Vidgren *et al.*, 2005;). The expression of *MPHx* genes seems to be strain specific. Expression was very low in several lager strains growing on maltose or glucose (Vidgren *et al.*, 2005), but Gibson *et al.* (2008) and James *et al.* (2003) found a stronger expression of *MPHx* during wort fermentations with other lager strains.

The rates of most enzyme-catalysed reactions approximately double for each 10 °C increase in temperature. However, reactions catalysed by integral membrane proteins usually exhibit nonlinear Arrhenius plots with increased temperature dependence at lower temperatures, where the structure of the membrane lipids changes from a more fluid liquid-crystalline phase to a more rigid gel phase. This phase transition depends on the membrane lipid composition (e.g. the presence and type of sterols and fatty acids) and on external factors, such as the osmotic pressure (Guyot *et al.*, 2006).

Rautio & Londesborough (2003) found strong temperature dependence for maltose transport (*c*. 70-fold between 0 and 20 °C) for an ale strain. However, Guimarães *et al.* (2006) reported a markedly smaller temperature dependence (*c*. 11-fold) for a lager strain. The apparent predominance of Agt1 transporters in ale strains, but not in lager strains, suggested that the different temperature dependencies of maltose transport (and, therefore, the fermentation rate) in ale and lager strains might reflect differences in the properties of their maltose transporters. This article presents evidence supporting this hypothesis. We report (1) the distribution of *MTT1*, defective *S. cerevisiae*-derived *AGT1* and *S. bayanus*-derived *AGT1* genes among ale and lager strains and (2) the temperature dependence of maltose transport into ale and lager strains and into genetically engineered yeasts expressing *AGT1, MALx1* and *MTT1* maltose transporter genes from particular brewer's yeast strains. A preliminary report of some of this work has been given (Vidgren *et al.*, 2007).

## Materials and methods

### Materials

U-^14^C maltose was from Amersham Biosciences (Espoo, Finland). Maltose for uptake experiments (minimum purity, 99%) and trehalose were from Sigma-Aldrich (Helsinki, Finland), and maltotriose was from MP Biomedicals (Solon, OH). Maltose and glucose for growth media were from Fluka (Helsinki, Finland). G418 was from Invitrogen (Espoo, Finland).

### Strains

The industrial strains used in this work are listed in Table 1. CMBS-33 was kindly provided by J.M. Thevelein (Katholieke Universiteit, Leuven, Belgium) and WS34/70 was from the Weihenstephan Brewery (Freising, Germany). The other strains were from the VTT Culture Collection. The frequently used strains A-63015, A-66024, A-75060 and A-10179 are hereafter referred to as A15, A24, A60 and A179, respectively. Two laboratory strains, CEN.PK2-1D (VW-1B; maltose-positive) and S150-2B (maltose-negative), were also used.

**Table 1:** Distribution of *AGT1, Sb-AGT1* and *MTT1* genes in some industrial yeasts

Strain	Origin	*AGT1*	Sb-AGT1*	*MTT1*
Lager strains				
A-60012	Weihenstephan 1	D	P	P
A-62013	Weihenstephan 294	D	P	P
A-63015 (A15)	Nordic brewery	D	P	P
A-66024 (A24)	Nordic brewery	D	P	P
A-82064	Nordic brewery	D	P	P
A-85072	Nordic brewery	D	P	P
A-95143	Nordic brewery	D	P	P
WS34/70	Weihenstephan	D	P	P
CMB33	Belgium	D	P	P
Ale strains				
A-10179 (A179)	UK brewery	P	M	M
A-60055	NCYC 1200	P		M
A-60056	NCYC 240	P		M
A-75060 (A60)	Nordic brewery	P	M	M
A-93116	NCYC 1087	P		M
Baker's yeasts				
B-62001	Nordic baker's yeast	P		P
B-62003	Nordic baker's yeast	P		P
Distiller's yeasts				
C-72051	Nordic distillery	P		P
C-77076	Nordic distillery	M		M
C-91180A	Nordic distillery	M		M

* Sb-AGT1 indicates a gene with 79% identity to *AGT1* recently discovered in WS34/70 by Nakao *et al.* (2009).

D, defective, frame shift mutation; P, present; M, missing.

### PCR analyses

Primers are shown in Table 2. PCR reactions were performed using standard procedures. To test for the presence of *MTT1* genes, total chromosomal DNA from each strain was used as a template with MTT1 Frw and MTT1 Rev primers to generate a 247-bp fragment. To test for the presence of *S. cerevisiae*-type *AGT1* genes, the primers AGT1 Frw and AGT1 Rev were used to generate 986-bp fragments. These fragments (842–1828 of the *AGT1* ORF) include the frame shift and premature stop codon (starting at nucleotide 1183) described previously (Vidgren *et al.*, 2005) in lager strains A15 and A24. They were cloned to a pCR-TOPO vector (Invitrogen) and sequenced using the AGT1Sekv4 primer to test for the frame shift. The putative *S. bayanus*-type *AGT1* gene (LBYG13187; Nakao *et al.*, 2009) was also studied. Most of the sequence of this gene has been published (Nakao *et al.*, 2009) and the 5′-terminal 400-bp sequence was kindly provided by Dr Y. Nakao. Total chromosomal DNA from several brewer's yeast strains and laboratory strain CEN.PK2-1D was used as a template with AGT1bay_Cl_Frw and AGT1bay_Cl_Rev primers to generate 1833-bp fragments corresponding to the complete ORF and stop codon. The fragments obtained were cloned into the pCR-TOPO vector and their sequences were determined using AGT1baySekv1–AGT1baySekv3 primers and universal M13 forward and reverse primers, which bind close to the cloning sites of pCR-TOPO.

**Table 2:** PCR primers

Name	Primer sequence*	Sequence detected†
MTT1_Cl_Frw	5′-CGAGATCTCGATGAAGGGATTATCCTCATT-3′	1–20 of *MTT1*
MTT1_Cl_Rev	5′-CGAGATCTCGTCATTTGTTCACAACAGATGG-3′	1828–1848 of *MTT1*
MTT1Sekv1	5′-CTTTGAATAGCAATACAG-3′	404–421 of *MTT1*
MTT1Sekv2	5′-AGAACTAGGATATAAGCT-3′	801–818 of *MTT1*
MTT1Sekv3	5′-TATCCAATATTGTCTTGG′3′	1212–1229 of *MTT1*
MTT1Sekv4	5′-GGTTATGTTTTGCCACTC-3′	1601–1618 of *MTT1*
MTT1Frw	5′-TTGGTAGGTTTGACCTTTAC-3′	1271–1290 of *MTT1*
MTT1Rev	5′-AGATGCCATATTATATGCGT-3′	1499–1518 of *MTT1*
AGT1Frw	5′-TTGCTTTACAATGGATTTGGC-3′	842–862 of *AGT1*
AGT1Rev	5′-CTCGCTGTTTTATGCTTGAGG-3′	1808–1828 of AGT1
AGT1Sekv4	5′-AAAGCAGATTGAATTGAC-3′	1011–1028 of *AGT1*
AGT1bay_Cl_Frw	5′-CGAGATCTCGATGAAAAATATACTTTCGCTGG-3′	1–22 of *Sb*-*AGT1*
AGT1bay_Cl_Rev	5′-GCAGATCTCGTCATAACGCCTGTTGACTCG-3′	1814–1833 of *Sb*-*AGT1*
AGT1baySekv1	5′-CCTACGATATCACTTCTC-3′	443–460 of *Sb*-*AGT1*
AGT1baySekv2	5′-CGCCTTACAATGGATCTG-3′	843–860 of *Sb*-*AGT1*
AGT1baySekv3	5′-ACGCTTGGTTCCTGGGTA-3′	1255–1272 of *Sb-AGT1*

* BglII restriction sites are underlined.

† The numbering is from the first nucleotide of the translational start. *Sb*-*AGT1* refers to the putative *Saccharomyces bayanus*-derived counterpart of *AGT1* (Nakao *et al.*, 2009).

### Laboratory strains bearing an ale or a lager strain *AGT1* gene in a multicopy plasmid

The *AGT1* genes from lager strain A15 and ale strains A60 and A179 were cloned by PCR using AGT1-F and AGT1-R primers. The sequences of these clones were verified as described earlier (Vidgren *et al.*, 2009). AGT1-F and AGT1-R primers bear BglII restriction sites, which facilitated the next cloning step, i.e., the ligation of the PCR fragments to YEplac195 multicopy vectors (Gietz & Sugino, 1988) at the BglII site between the *PGK1* promoter and terminator. In addition, the *KanMX* cassette (Wach *et al.*, 1994) was introduced into the YEplac195 plasmid at the multiple cloning site to confer resistance to G418. The laboratory strain S150-2B was transformed with these YEplac195-*PGK1*-*AGT1-KanMX* constructs or with the empty YEplac195-*KanMX* plasmid as a control. The lithium acetate transformation procedure (Gietz *et al.*, 1992) was used and transformants were selected using G418 selection.

### Laboratory strains bearing a lager strain *MTT1* or *MALx1* gene in a multicopy plasmid

*MTT1* and *MALx1* genes were cloned from lager strain A15 by PCR with standard procedures using MTT1 Cl Frw and MTT1 Cl Rev primers, which contain BglII sites. Because the sequences of the *MTT1* and *MALx1* ORFs are identical to each other at both their starts and their ends, both genes were obtained with these primers. The PCR products were cloned into the pCR-TOPO vector and their sequences were determined using MTT1Sekv1–MTT1Sekv4 primers and universal M13 forward and reverse primers, which bind near the cloning site of pCR-TOPO. From the nine sequenced clones, four were >99% identical to the *MTT1* sequence reported by Dietvorst *et al.* (2005) and five were >98% identical to the *MAL31* type sequence in the SGDB. Clone 1 was 100% identical to the *MTT1* sequence of Dietvorst *et al.* (2005) and was chosen to represent *MTT1*. Clone 2 was 99% identical to the *MAL31* sequence in the SGDB and was chosen to represent *MALx*1. They were excised from the pCR-TOPO plasmid using the BglII enzyme and ligated between the *PGK1* promoter and terminator at the BglII site in the YEplac195 multicopy vector. The laboratory strain S150-2B was transformed with either the YEplac195-*PGK1*-*MTT1* or the YEplac195-*PGK1*-*MALx1* construct using the lithium acetate transformation procedure (Gietz *et al.*, 1992).

### Construction of a lager yeast with an integrated, ale yeast-type *AGT1* gene

Construction of Integrant 1 has been described previously (Vidgren *et al.*, 2009). Briefly, the defective *AGT1* gene in the lager strain A15 (with a premature stop codon at nucleotide 1183) was repaired using an integration cassette containing nucleotides 1–1478 of the *AGT1* ORF from ale strain A60 functionally fused to a *PGK1* promoter and flanked on the 5′-side by the *AGT1* promoter sequence (−1 to −705). The ORF of the repaired gene has the ale yeast sequence from nucleotide 1 to somewhere between 1183 and 1478 (i.e. the frame shift and premature stop codon are removed), followed by the lager yeast sequence to the end of the *AGT1* gene, and it is under the control of a *PGK1* promoter.

### Maltose transport assays

For maltose transport studies, native ale and lager strains were grown in YP (10 g yeast extract and 20 g peptone L^−1^) containing 40 g maltose L^−1^. YP-40 g glucose L^−1^ was used for the growth of Integrant 1, so that its endogenous maltose transporters were repressed. YP-40 g glucose L^−1^ supplemented with G418 (200 mg L^−1^) was used for S150-2B derivatives transformed with a YEplac195-*KanMX* plasmid (with or without an *AGT1* gene). S150-2B derivatives transformed with YEplac195 plasmids lacking *KanMX* (and with or without an *MTT1* or *MALx1*) were grown in a synthetic complete medium (Sherman *et al.*, 1983) lacking uracil and containing 20 g glucose L^−1^. Yeasts were grown in 100 mL of medium in 250-mL Erlenmeyer flasks at 150 r.p.m. and 24 °C. They were usually harvested at an OD_600 nm_ between 4 and 7 (i.e. at 2±1 mg dry yeast mL^−1^) while sugar was still present, but were grown into the stationary phase when so stated. After centrifugation (10 min, 9000 ***g***, 0 °C), the yeast pellets were washed with ice-cold water and then with ice-cold 0.1 M tartrate-Tris (pH 4.2) and finally suspended in the same buffer to 200 mg of fresh yeast mL^−1^. For standard assays, about 1-mL portions of yeast suspension were equilibrated for 10 min to assay temperature (0–20 °C) in a water bath. Zero-*trans* [^14^C]-maltose uptake rates were then determined at 5 mM maltose (unless stated otherwise) as described (Guimarães *et al.*, 2006). Reactions were started by adding 40 μL of yeast suspension to 20 μL containing 15 mM [^14^C]-maltose (about 1000 c.p.m. nmol^−1^) and any inhibitors specified in the text. Reactions were stopped after 10–300 s by addition of 10 mL ice-cold water and immediate filtration through a (prewashed) HVLP membrane (Millipore). The membrane was rinsed with another 10 mL of ice-cold water and transferred to a scintillation cocktail and the radioactivity in the trapped yeast was counted. To ensure linearity with respect to time, two reaction times, *t* and 2*t*, were used, each in duplicate. Reaction times were chosen according to the yeast and temperature, so that the reaction rate calculated from the 2*t* assays was at least 90% of that calculated from the *t* assays.

### Stimulation of maltose transport by glucose

**Fig. 1:**
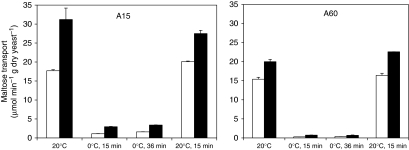
Temperature dependence of maltose transport by lager (A15) and ale (A60) strains. Yeasts were harvested during growth on maltose at 24°C and their maltose transport activities were assayed at 20 and 0°C. For standard assays (white columns), the yeasts were equilibrated to 20°C for 8 min and then assayed at 20°C, transferred to 0°C and assayed at 0°C after 15 and 36 min and then returned to 20°C and reassayed at 20°C after 15 min. For glucose-activated assays (black columns), the same procedure was used, except that after 4 min at 20°C, glucose was added to 28 mM and incubation was continued for 8 min before the first 20°C assay. Results are averages±ranges of duplicate assays.

Where indicated, yeast suspensions were treated with glucose immediately before the maltose transport assays as described by Guimarães *et al.* (2008). The yeast suspension was mixed with 0.1 volume of 0.28 M glucose, incubated for 8 min at 20 °C and then maltose transport was assayed as described above. For the study of Fig. 1, the glucose-treated yeast was first assayed at 20 °C, then immediately transferred to an ice-water bath and assayed at 0 °C after 15 min and again after 36 min. The yeast suspension was then immediately returned to a 20 °C bath and assayed after a further 15 min.

## Results

### Functionality and distribution of *S. cerevisiae*-type *AGT1* genes among yeast strains

The *AGT1* maltose transporter genes in two ale strains (A60 and A179) differ slightly from the sequence in the SGDB, but encode full-length proteins, whereas those in two lager strains (A15 and A24) encode truncated, 394 amino acid polypeptides because of a frame shift and a premature stop codon at nucleotide 1183 (Vidgren *et al.*, 2005). To determine the functionality of these genes, the laboratory strain S150-2B (maltose-negative) was transformed with plasmids containing *AGT1* genes from A15, A60 or A179 under *PGK1* promoters or with the empty plasmid. Transformants were grown on glucose, harvested in the early stationary phase and assayed for maltose transport. Transformation with the empty plasmid or with *AGT1* from lager strain A15 did not increase transport activity. Transformation with *AGT1* from ale strains increased the maltose transport activity from <0.2 U g^−1^ dry yeast (control plasmid) to 13.1±0.4 U g^−1^ dry yeast (*AGT1* from A60; mean±SD, *n*=3) or 10.0 U g^−1^ dry yeast (*AGT1* from A179). Maltose transport by transformants carrying *AGT1* genes from A60 or A179 was strongly inhibited by 50 mM maltotriose (85% and 79%, respectively) and 75 mM trehalose (94% and 85%, respectively), which is characteristic of maltose transport by the broad specificity Agt1 transporter. Between 0.5 and 55 mM maltose, the transporter encoded by *AGT1* from A60 exhibited a single *K*_m_ of 1.5 mM maltose, which is lower than that (5–10 mM) estimated by Han *et al.* (1995) and much lower than that reported (18 mM) by Stambuk & de Araujo (2001). These results show that the *AGT1* genes from these two ale strains encode functional, broad-specificity α-glucoside transporters, whereas the defective *AGT1* gene from lager strain A15 does not encode a functional maltose transporter.

The distribution of *AGT1* genes in different kinds of industrial yeasts was studied by PCR (Table 1). All nine lager strains studied contained *S. cerevisiae*-type *AGT1* genes with the same defect as strains A15 and A24. All five ale strains, both baker's strains and one of the three distiller's strains studied contained *AGT1* genes without this defect. The other two distiller's strains lacked *AGT1*. It can be concluded that this particular *AGT1* gene mutation, producing a premature stop codon, is characteristic of lager strains. These studied lager strains are not, to our knowledge, more closely related to each other than are lager strains in general.

### Distribution of an *S. bayanus*-type *AGT1* gene in brewer's yeast strains

The *Sb-AGT1* gene (Nakao *et al.*, 2009) is only 79% identical at the nucleotide level to *AGT1* from *S. cerevisiae*, and so might not be revealed by earlier Southern hybridization and PCR studies using probes and primers designed for *AGT1* from *S. cerevisiae* (Jespersen *et al.*, 1999; Vidgren *et al.*, 2005;). Using primers designed for *Sb-AGT1*, we found this gene in all the lager strains studied, but not in either studied ale strain (Table 1) or in the maltose-positive laboratory strain CEN.PK2-1D. In two tested lager strains, A15 and A24, the sequence of the *Sb-AGT1* gene was 100% identical to that reported by Nakao *et al.* (2009) for *Sb-AGT1* of WS34/70, which encodes a polypeptide of 610 amino acids.

### Distribution of *MTT1* genes in ale and lager strains

*MTT1* genes have earlier been demonstrated in lager strains PYCC4457 (the type strain of *S. carlsbergensis*) (Salema-Oom *et al.*, 2005) and A15, CMBS33, OG2252 and WS34/70 (Dietvorst *et al.*, 2005). We found *MTT1* in all nine tested lager yeast strains (including three of the above-mentioned ones), but not in any of the five ale strains (Table 1). An *MTT1* gene was also present in both tested baker's strains and in one of the three distiller's strain, the same that also contained an *AGT1* gene.

### The temperature dependence of maltose uptake by brewer's yeast strains

Brewer's yeasts were harvested during growth on maltose at 24 °C and their maltose transport activities were assayed at different temperatures. For lager strain A15, the activity measured in the standard way at 0 °C was 9.3±0.9% of that at 20 °C, whereas for ale strain A60, the activity at 0 °C was 2.1±0.1% of that at 20 °C (Fig. 1, open columns).

Maltose transport is active and depends on the transmembrane electrochemical potential. When yeast cells growing on fermentable sugar are harvested, washed and suspended in a medium lacking a carbon source, their intracellular adenylate energy charge (and therefore their membrane potential) can decrease. The adenylate energy charge and maltose transport rates of such cells can be increased by treatment with glucose for a few minutes immediately before the zero-*trans* maltose uptake assay (Guimarães *et al.*, 2008). This activation with glucose increased the maltose transport activity of both A15 and A60, but did not eliminate the difference in temperature sensitivity between the two yeasts. For glucose-activated A15, the maltose transport rate at 0 °C was 10.9±1.4% of that at 20 °C, and for glucose-activated A60, it was 3.4±0.3% (Fig. 1, black columns). These results showed that there was a difference between the temperature sensitivities of maltose transport by the lager and ale strains that could not be explained by differences in adenylate energy charge. In further work, standard assays, without glucose activation, were used.

**Fig. 2:**
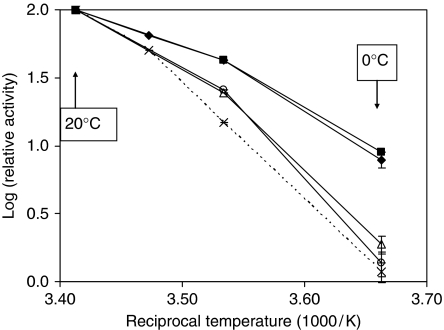
Arrhenius plots of maltose transport by the lager strains A15(♦) and A24(▪), ale strains A179(○) and A60(Δ) and the strain Integrant 1 (×). For each data set, rates are expressed as percentages of the rate at 20°C. Absolute rates (μmol min^−1^ g^−1^ dry yeast at 20°C) varied between 12 and 27 for A15, A24, A60 and A179 and between 2 and 4 for Integrant 1. Results at 0°C are means±SDs for A15 (*n*=4) and A60 (*n*=3) and means±ranges of independent duplicates for A179 and Integrant 1.

When harvested during growth on maltose, two lager strains and two ale strains had similar maltose transport activities at 20 °C, but the activities of both ale strains were markedly more temperature dependent than those of the lager strains (Fig. 2). At 20 °C, the difference between the lager and ale strains was not significant (in μmol min^−1^ g^−1^ dry yeast, 20.3±3.5 for the lager strains and 19.2±5.9 for the ale strains; means±SDs, *n*=5, *P*>0.72 (two-tail Student's *t*-test). However, at 0 °C, the lager strains had about fivefold greater activity than the ale strains and the difference was highly significant. Maltose transport activities at 0 °C in μmol min^−1^ g^−1^ dry yeast were 1.7±0.4 (8.4% of the 20 °C activity) for the lager strains and 0.31±0.05 (1.6% of the 20 °C activity) for the ale strains (means±SDs, *n*=5; *P*<0.002). The relatively smaller activities of the ale strains were also evident at 10 °C.

### The temperature dependence of an Agt1-type transporter

Integrant 1 is a derivative of lager strain A15 containing a chimeric *AGT1* in place of the defective native *AGT1* of A15. The chimera consists of nucleotides 1 to *x* (where *x* is between 1183 and 1478) of an *AGT1* gene from ale strain A60 and nucleotides *x*+1 to 1848 of the native *AGT1* of strain A15, driven by a *PGK1* promoter (Vidgren *et al.*, 2009). It encodes a functional, 616 amino acid Agt1 transporter, with the same amino acid sequence as Agt1 of strain A60, because after nucleotide *x*, the ale and lager versions of *AGT1* encode the same amino acid sequence (Vidgren *et al.*, 2005). Compared with A15, Integrant 1 has considerably increased maltose and maltotriose transport activity during growth on glucose (when A15 has negligible activities) and slightly increased maltose transport activity, but considerably increased maltotriose transport activity during growth on maltose (Vidgren *et al.*, 2009). Thus, Integrant 1 produces a functioning Agt1 transporter in a lager yeast background. When grown on glucose, this Agt1 is expected to be the only maltose transporter present (because glucose-grown A15 lacks maltose transport activity). The temperature dependence of maltose transport by glucose-grown Integrant 1 was much greater than that of the lager strains and at least as great as that of the ale strains (Fig. 2).

### The temperature dependence of Malx1 and Mtt1 transporters

**Fig. 3:**
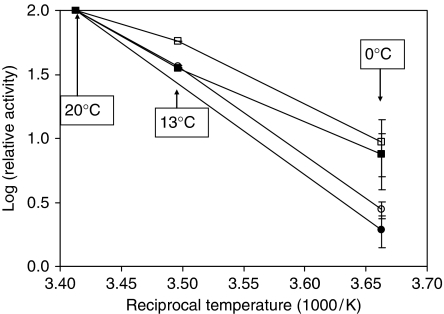
Arrhenius plots of maltose transport by Mtt1 and Malx1 transporters. Maltose transport was measured using S150-2B transformed with *MTT1* (□, ▪) or *MALx1* (○, •). The maltose concentration in the transport assay was 5 mM (□, ○, •) or 50 mM (▪) and transformants were harvested during growth on glucose (at OD_600 nm_ of 3–5; □, ▪, ○) or in the stationary phase (OD_600 nm_ of 10; •). Rates at 13 and 0°C are expressed as percentages of the rate at 20°C. Values at 0°C are (□, ○) means±SDs (*n*=4 or 5) or (▪, •) means±ranges of duplicate experiments.

*MALx1* (99% identical to *MAL31* in the SGDB) and *MTT1* (100% identical to the sequence reported by Dietvorst *et al.*, 2005) were cloned from lager strain A15. The maltose-negative laboratory yeast, S150-2B, was transformed with plasmids containing these genes under the control of *PGK1* promoters. Untransformed S150-2B had negligible maltose transport activity (<0.2 μmol min^−1^ g^−1^ dry yeast at 20 °C). *MALx1* transformants had high activity (55 μmol min^−1^ g^−1^ dry yeast) during growth on glucose and lower activity (6 μmol min^−1^ g^−1^ dry yeast) in the stationary phase. Both growing- and stationary-phase transformants exhibited strong temperature dependence (Fig. 3). The rates at 0 °C compared with 20 °C were 2.8±0.4% (mean±SD, *n*=5) for growing cells and 1.9±0.6% (mean±range; *n*=2) for stationary-phase cells.

Compared with *MALx1* transformants, both growing and stationary-phase *MTT1* transformants exhibited lower maltose transport activity at 20 °C, about 1.0 μmol min^−1^ g^−1^ dry yeast at 5 mM maltose and 6.5 μmol min^−1^ g^−1^ dry yeast at 50 mM maltose. Mtt1 is reported to have a high *K*_m_ for maltose (60–90 mM, Salema-Oom *et al.*, 2005; 40 mM, Multanen, 2008). At both maltose concentrations, the transport activity exhibited relatively small temperature dependence (Fig. 3). Activities at 0 °C compared with 20 °C were 9.4±4.4% (5 mM; mean±SD, *n*=4) and 7.5±2.5% (50 mM; mean±range, *n*=2). The absolute maltose transport activity at 50 mM maltose (6.5 μmol min^−1^ g^−1^ dry yeast) was similar to that at 5 mM maltose of stationary-phase cells transformed with *MALx1* (6 μmol min^−1^ g^−1^ dry yeast). Thus, the marked differences in temperature dependence between cells transformed with *MALx1* and cells transformed with *MTT1* are not explained by differences in their absolute maltose transport activities.

## Discussion

Because Agt1 is the only maltose transporter known to accept both trehalose and α-methyl glucoside as substrates (Day *et al.*, 2002b; Salema-Oom *et al.*, 2005;), the strong inhibition of maltose transport by both trehalose and α-methyl-glucoside in ale strains, but not lager strains (Rautio & Londesborough, 2003; Vidgren *et al.*, 2005;), suggests that Agt1 transporters are the dominant maltose transporters in ale strains, but not lager strains. Vidgren *et al.* (2005) showed that two lager strains, A15 and A24, contain *AGT1* genes with a premature stop codon starting at nucleotide 1183. Here, we show that the *AGT1* from A15 does not encode a functional maltose transporter. Nakao *et al.* (2009) also found this premature stop codon in an *AGT1* gene in a third lager strain, WS34/70. We extend these results to show that the premature stop codon at 1183 is present in the *S. cerevisiae*-type *AGT1* genes of all nine tested lager strains, but is absent from all five tested ale strains. Because the premature stop codon is caused by an easily reversible point mutation, then if the Agt1 transporter were advantageous to lager strains in their normal habitat, one would expect to find lager strains in which this reversal has occurred. The observations that reversal has not occurred in any of the nine tested lager strains, whereas the mutation causing the premature stop codon was not present in any of the five ale strains, suggest that there has been selection pressure in favour of inactivation of *AGT1* during the evolution of lager strains, but not during the evolution of ale strains. The main difference between the conditions under which ale and lager strains have evolved is the lower temperature of lager fermentations. *MTT1* genes were present in all nine lager strains, but in none of the five ale strains. This suggests that the replacement of Agt1 transporters by Mtt1 transporters is an important difference between lager and ale strains, probably related to the lower temperature of lager fermentations.

We show that maltose transport is more strongly temperature dependent in two tested ale strains than in two tested lager strains (the yeasts were grown at 24 °C and then assayed at different temperatures). At 20 °C, all four strains had similar maltose transport activity, but at 0 °C, the ale strains showed about fivefold smaller activities. When single maltose transporters were studied, using genetically engineered strains, their temperature dependence decreased in the order Agt1≥Malx1>Mtt1. The temperature dependence of Mtt1 (in a laboratory strain) was similar to that of maltose transport by lager yeasts. An ale-type Agt1 transporter working in a lager yeast (Integrant 1) had the high temperature dependence of maltose transport observed for ale yeasts (Fig. 2). This suggests that the different temperature dependencies of maltose transport by ale and lager yeasts result from the different maltose transporters present in these yeasts rather than, for example, a hypothetical difference in the lipid composition of their plasma membranes. Thus, the Agt1 transporter in Integrant 1 had been inserted into a lager yeast membrane, but still exhibited high temperature dependence, which therefore was a property of the transporter protein itself rather than a property of the lipid membrane of ale strains.

We do not yet know what differences between Agt1 and Mtt1 transporters account for their different temperature dependencies. Membrane proteins are sensitive to membrane lipid composition and dynamics. They can have specific lipid requirements for their optimal activity (Opekarová & Tanner, 2003), correct orientation of transmembrane helices (Bogdanov *et al.*, 2002), targeting to the yeast plasma membrane (Umebayashi & Nakano, 2003; Toulmay & Schneiter, 2007;) and stable localization in the plasma membrane (Mitsui *et al.*, 2009). The high temperature dependence of reactions catalysed by enzymes embedded in lipid membranes may result from work carried out by the enzyme on surrounding lipid as a result of changes in protein shape during the catalytic cycle (Londesborough, 1980). Thus, one possibility is that Agt1 exhibits greater shape changes than Mtt1 during the catalytic cycle, and so performs more work on the surrounding lipid membrane. Nakao *et al.* (2009) found seven α-glucoside transporter genes in the genome of lager yeast WS34/70, two of which, *S. bayanus-*derived *MALx1* and *S. cerevisiae-*derived *AGT1*, encoded truncated proteins. They also noted an increase in the copy number of *MTT1*, which was present on both the *S. bayanus* and the *S. cerevisiae* versions of chromosome VII. These results are consistent with the suggestion that in lager strains, Mtt1 transporters have become more important at the expense of Agt1 and Malx1 transporters. Nakao *et al.* (2009) located a *MAL31* gene to *S. cerevisiae* chromosome II (Chr. Sc-II) and an *MPH2* gene to Chr. Sc-IV. These loci were earlier observed in most, but not all, studied lager strains by hybridization of specific probes to chromosome blots (Jespersen *et al.*, 1999; Vidgren *et al.*, 2005;). These hybridization studies found binding of a *MAL61*-probe (expected to recognize all *MALx1* genes) to Chr. VII from both lager and ale strains, whereas Nakao *et al.* (2009) found an *MTT1* gene on both the *S. cerevisiae* and the *S. bayanus* versions of Chr. VII from lager strain WS34/70. *MTT1* is 91% identical to *MALx1*, and so probably in the lager strains, the *MAL61-*probe bound to *MTT1* genes, which were not known at the time of these hybridization studies. Nakao *et al.* (2009) found a truncated *S. cerevisiae*-derived *AGT1* gene, but did not locate the gene. Presumably, this is the *AGT1* detected on Chr. VII in both hybridization studies. Nakao *et al.* (2009) also reported an *S. bayanus*-derived *AGT1* (*Sb-AGT1*) on Chr. Sb-XV-VIII and a truncated, *S. bayanus*-derived *MAL31* on Chr. Sb-V. Neither of these genes was noted in the hybridization studies, which used probes based on *S. cerevisiae* sequences. Our present results show that *Sb-AGT1* is present in all eight studied lager strains, but, as expected, in neither of the two ale strains studied. The sequence of the *Sb-AGT1* in strains A15 and A24 was identical to that reported by Nakao *et al.* (2009). No information is available on the catalytic properties of the transporter encoded by this gene (it is only 79% identical to the *AGT1* from *S. cerevisiae*).

Both hybridization studies detected *MAL21* and *MAL41* genes on Chr. III and Chr. XI, respectively. Such genes were not observed by Nakao *et al.* (2009) in Weihenstephan 34/70. The estimated sequence coverage was 95.8%, and so one or both genes might be in the unsequenced 4.2%. Alternatively, Weihenstephan 34/70 may lack these genes. Hybridization indicated that *MAL21* was lacking from 13 of the 25 studied lager strains and *MAL41* was lacking from one lager strain (Jespersen *et al.*, 1999). Both our present results and those of Nakao *et al.* (2009) show that in lager strains, the number of different functional α-glucoside transporters is less than the number of different α-glucoside transporter (pseudo)genes. Inactivation of genes encoding less suitable transporters seems to be one way in which lager strains have evolved for low-temperature fermentations.

## Statement

Re-use of this article is permitted in accordance with the Terms and Conditions set out at http://www3.interscience.wiley.com/authorresources/onlineopen.html
